# A novel deep learning model based on multimodal contrast-enhanced ultrasound dynamic video for predicting occult lymph node metastasis in papillary thyroid carcinoma

**DOI:** 10.3389/fendo.2025.1634875

**Published:** 2025-07-24

**Authors:** Rongwei Liu, Fengqin Yuan, Biaoyang Wang, Weihua Chen, Jun Ye, Yun He

**Affiliations:** ^1^ Department of Medical Ultrasound, The First Affiliated Hospital of Gannan Medical University, Ganzhou, Jiangxi, China; ^2^ Department of Medical Ultrasound, The First Affiliated Hospital of Guangxi Medical University, Nanning, Guangxi Zhuang Autonomous Region, China

**Keywords:** papillary thyroid carcinoma, occult lymph node metastasis, dynamic video, deep learning, contrast-enhanced ultrasound

## Abstract

**Objective:**

This study aimed to evaluate the value of constructing a multimodal deep-learning video model based on 2D ultrasound and contrast-enhanced ultrasound (CEUS) dynamic video for the preoperative prediction of OLNM in papillary thyroid carcinoma (PTC) patients.

**Methods:**

A retrospective analysis was conducted on 396 cases of clinically lymph node-negative PTC cases with ultrasound images collected between January and September 2023. Five representative deep learning architectures were pre-trained to construct deep learning static image models (DL_image), CEUS dynamic video models (DL_CEUSvideo), and combined models (DL_combined). The area under the receiver operating characteristic curve (AUC) was used to evaluate model performance, with comparisons made using the Delong test. A P-value of less than 0.05 was considered statistically significant.

**Results:**

The DL_CEUSvideo, DL_image, and DL_combined models were successfully developed and demonstrated. The AUC values were 0.826 (95% CI: 0.771-0.881), 0.759 (95% CI: 0.690-0.828), and 0.926 (95% CI: 0.891-0.962) in the training set, and 0.701 (95% CI: 0.589-0.813), 0.624 (95% CI: 0.502-0.745), and 0.734 (95% CI: 0.627-0.842) in the test set. Finally, sensitivity, specificity, and accuracy for the DL_CEUSvideo, DL_image, and DL_combined models were 0.836, 0.671, 0.704; 0.673, 0.716, 0.707; and 0.818, 0.902, 0.886 in the training set, and 0.556, 0.775, 0.724; 0.556, 0.674, 0.647; and 0.704, 0.663, 0.672 in the test set, respectively.

**Conclusion:**

These results demonstrated that the multimodal deep learning dynamic video model could preoperatively predict OLNM in PTC patients. The DL_CEUSvideo model outperformed the DL_image model, while the DL_combined model significantly enhanced sensitivity without compromising specificity.

## 1 Introduction

Papillary thyroid carcinoma (PTC) is the most prevalent pathological type of malignant thyroid tumors, accounting for approximately 84% of cases, with its incidence rising globally (1). Earlier studies have established that despite the generally favorable prognosis of PTC patients, approximately 30%–65% of patients experience occult lymph node metastasis (OLNM) (2–4), which has been established as a risk factor for postoperative local recurrence and distant metastasis that directly affects preoperative surgical decision-making (3, 5, 6). More importantly, the lack of preoperative imaging and clinical evidence for OLNM poses challenges in detecting OLNM preoperatively. At present, OLNM is primarily diagnosed in clinically lymph node-negative PTC patients through prophylactic central lymph node dissection (CLND) (7). However, CLND increases the risk of surgical complications, such as recurrent laryngeal nerve injury and hypocalcemia (8), leading to debate over the necessity of prophylactic lymph node dissection (9–12). Therefore, there is a pressing need to develop non-invasive and accurate preoperative methods for predicting OLNM to optimize treatment strategies and individualize prognostic assessment in PTC patients.

The 2015 American Thyroid Association guidelines recommend preoperative ultrasound examination to initially assess lymph node metastasis in PTC patients (9). However, the anatomical complexity of central lymph nodes poses challenges in routine ultrasound, making it difficult to identify OLNM preoperatively (13, 14). Numerous studies have concluded that conventional ultrasound features of PTC, such as tumor size, location, microcalcifications, and extrathyroidal extension, are closely related to OLNM (4, 15, 16). Noteworthily, the advent of Contrast-Enhanced Ultrasound (CEUS) in thyroid imaging has introduced features such as peak enhancement intensity, enhancement direction, presence of ring enhancement, and enhancement components to offer additional diagnostic information for OLNM in PTC patients (17, 18). However, these ultrasound features typically rely on the examiner’s subjective judgment, lacking objective predictive indicators.

Artificial intelligence (AI) excels in the quantitative evaluation of imaging data, demonstrating significant potential in assisting physicians to achieve more accurate and reproducible results. In recent years, deep learning (DL) has garnered widespread attention for its outstanding performance in medical image recognition tasks. For instance, it can effectively enhance the accuracy of medical image interpretation, thereby increasing the objectivity of disease diagnosis (19–21). Previous studies (22–26) have primarily focused on generating deep-learning models using single-frame ultrasound images to predict lymph node metastasis. However, both lymph node lesions and primary PTC lesions exhibit heterogeneity, and single-frame static images fail to comprehensively capture their features, leading to the loss of critical tumor characteristics. Utilizing CEUS dynamic video to construct deep learning models for predicting OLNM can partially address this shortcoming. Previous studies (27–30) have demonstrated that DL models incorporating CEUS have achieved favorable performance in predicting microvascular invasion (MVI) of hepatocellular carcinoma (HCC), identifying high-risk patients for early postoperative HCC recurrence, differentiating pancreatic ductal adenocarcinoma from chronic pancreatitis, and assessing the vulnerability of carotid atherosclerotic plaques. At present, multimodal DL models integrating conventional two-dimensional ultrasound and CEUS dynamic video have not been applied to predict OLNM in PTC. At present, multimodal deep learning models integrating conventional two-dimensional ultrasound and CEUS dynamic video have not been applied to predict OLNM in papillary thyroid carcinoma. Thus, the present study aimed to evaluate the value of a multimodal deep learning video model constructed from preoperative two-dimensional ultrasound and CEUS dynamic video of PTC primary lesions for predicting OLNM in clinically lymph node-negative PTC patients.

## 2 Materials and methods

### 2.1 Study design and patients

**Figure 1:**
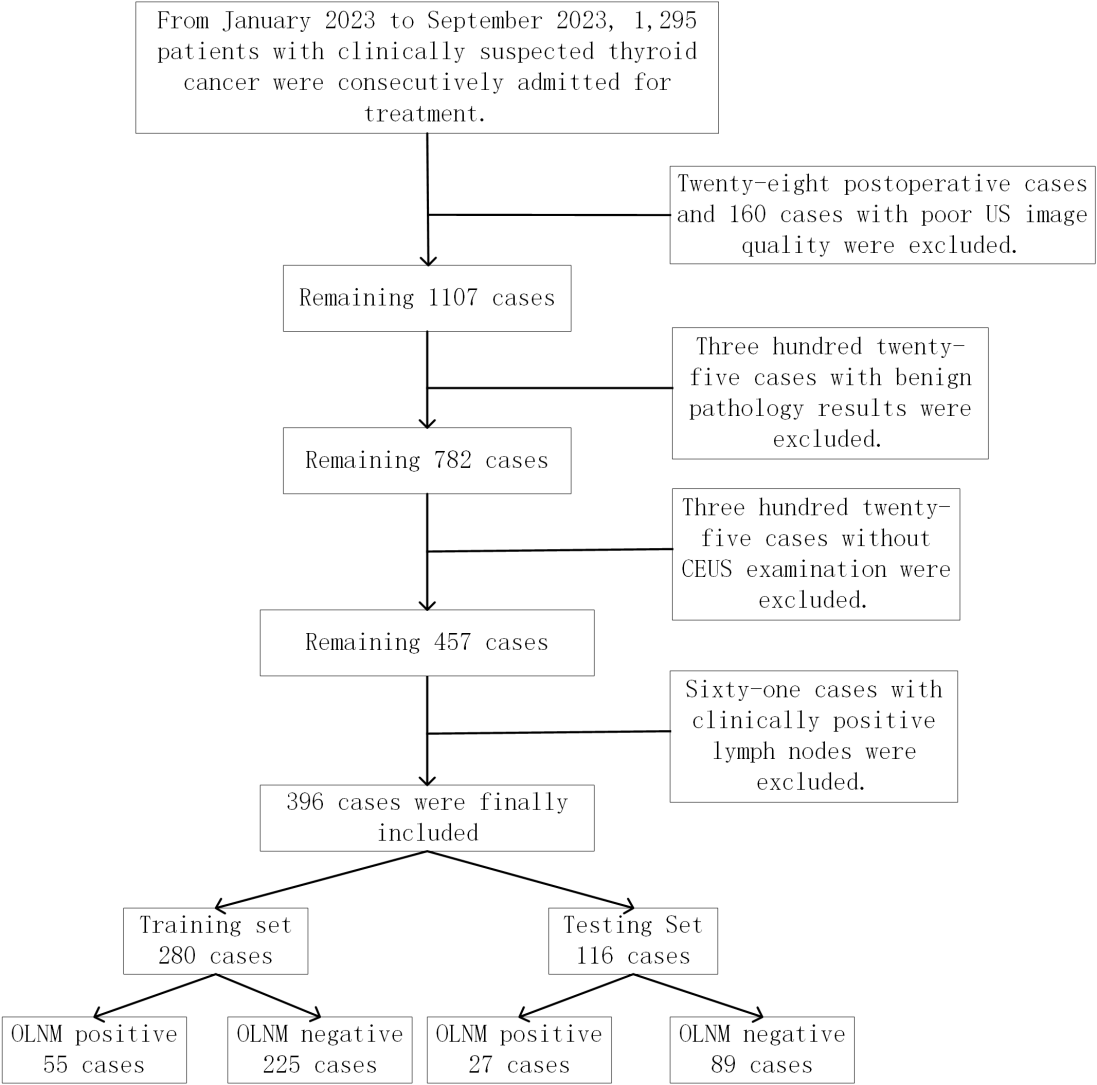
Patient recruitment and grouping for the study. A total of 396 patients with papillary thyroid carcinoma from two medical centers between January 2023 and September 2023 were enrolled in our study. These patients were divided into a training set and a test set, with 280 in the training set and 116 in the test set.

This retrospective study was approved by the Institutional Ethics Review Board (2024-E0890), and the requirement for informed consent was waived. Between January 2023 and September 2023, ultrasound images from 396 clinically lymph node-negative PTC patients were acquired from the thyroid ultrasound image database at the First Affiliated Hospital of Guangxi Medical University (center 1) and First Affiliated Hospital of Gannan Medical University (center 2). These patients were divided into a training set and a test set, with 280 in the training set and 116 in the test set. Inclusion criteria: 1. Patients who underwent surgical intervention and lymph node dissection; 2. Postoperative pathological diagnosis of unifocal PTC; 3. Underwent routine ultrasound and CEUS within one month before surgery; 4. No lymph node abnormalities on preoperative clinical and neck ultrasound. Exclusion criteria: 1. History of previous surgery or ablation; 2. Incomplete or poor-quality ultrasound images; 3. Lack of CEUS examination; 4. Postoperative pathology indicating benign nodules. The study inclusion flowchart is illustrated in **Figure 1**.

### 2.2 Ultrasound and contrast-enhanced ultrasound examinations

The ultrasound equipment used in this study included GE LOGIQ E9, Mindray Resona 7, and Philips EPIQ7. Routine ultrasound and CEUS were performed within one month prior to surgical intervention. Informed consent was obtained from each patient before CEUS examination. Patients were positioned in a supine position, maintained normal breathing, and were instructed to avoid swallowing. Initially, the L14–5 probe was used to scan the suspicious thyroid nodule from multiple angles, and the largest plane was selected for CEUS (CEUS parameters are shown in the **Supplementary Material**).

### 2.3 Image preprocessing

Following the completion of routine two-dimensional ultrasound and CEUS examinations, the highest-quality video recording and single-frame images with minimal artifacts were selected for analysis. All thyroid ultrasound dynamic videos extracted from the system were converted and stored in Audio Video Interleave (AVI) format. Upon obtaining the raw thyroid CEUS dynamic video data, the nodule region was extracted. Next, a physician with over five years of experience in thyroid CEUS reviewed the video to determine nodule size and boundaries, ensuring image quality. Thyroid CEUS videos (lasting approximately 10–120 seconds) were downsampled at a rate of one frame per two seconds to extract keyframes. For each video, 1–15 frames with clear nodule features were selected, and the final five keyframes were selected at equal time intervals. This method reduced the need for ultrasound specialists to delineate all nodule regions in the video, as minimal variation exists between adjacent frames within a two-second interval, allowing efficient capture of key nodule features. The downsampling rate for video keyframe extraction and the selection of five temporally equidistant peak frames were empirically determined based on the current study.

Thereafter, the extracted video data images were preprocessed and normalized as follows: 1. Only the central lesion area was retained by cropping and excluding irrelevant edge information; 2. Ultrasound images were resized to 256×256 pixels, following which data augmentation methods such as random flipping and cropping were applied to enhance data diversity, model generalization, and robustness, thereby mitigating the risk of overfitting; 3. Ultrasound images were scaled to 224×224 pixels, and pixel values were normalized to a range of 0 to 1.

**Figure 2:**
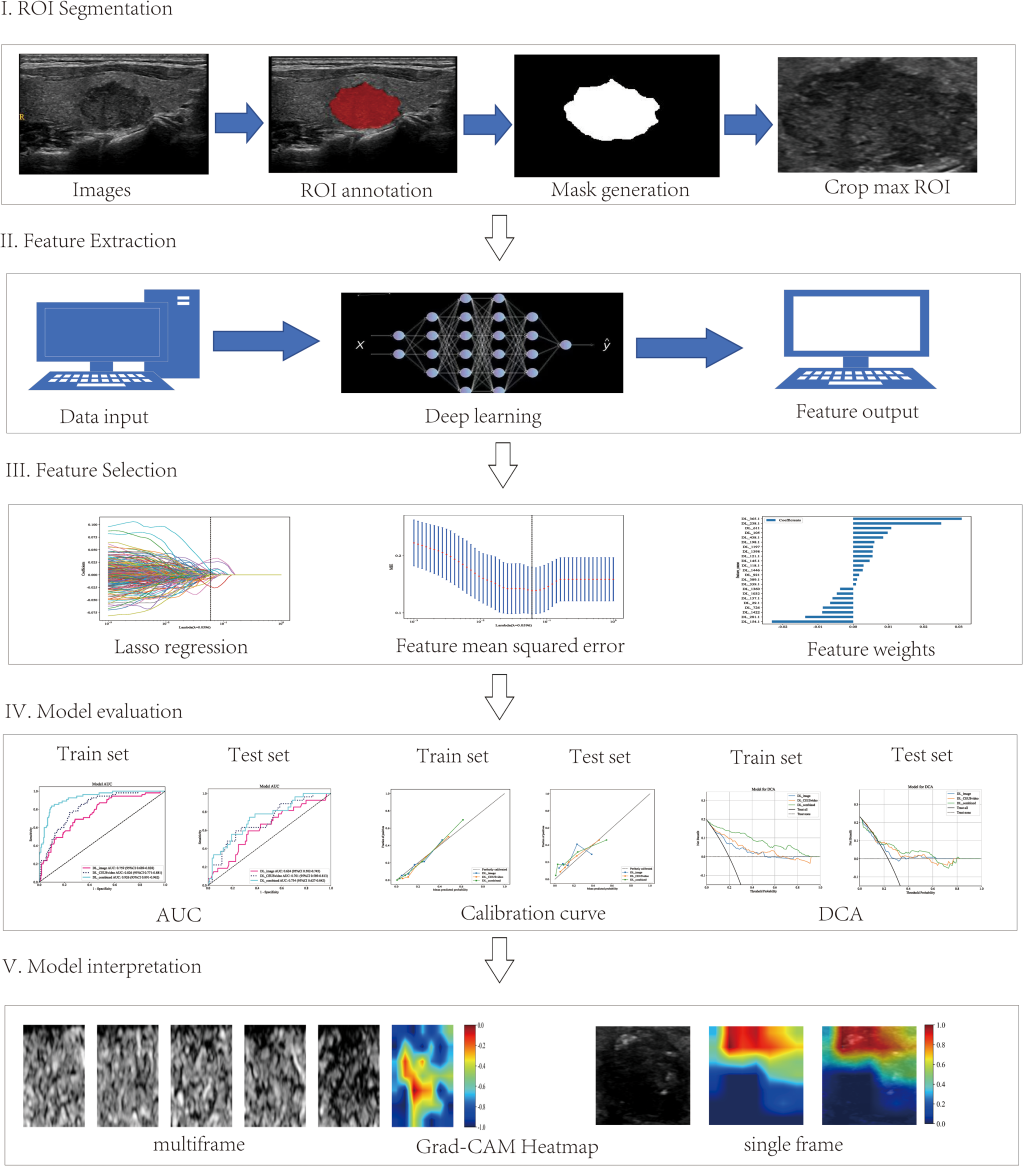
Workflow of the deep learning analysis. The workflow includes tumor segmentation, feature extraction, feature selection, model evaluation, and clinical implications of the interpretable deep learning signature.

Afterward, the preprocessed ultrasound images were loaded into ITK-SNAP software (version 3.8.0) for manual segmentation and precise annotation of nodule regions. Lastly, the masks were saved for subsequent model training and feature extraction. The detailed workflow is displayed in **Figure 2**.

### 2.4 Development of 2D static images, CEUS dynamic video, and DL_combined models

A total of five representative deep learning architectures, namely DenseNet121, DenseNet169, DenseNet201, ResNet18, and ResNet34, were employed for model pre-training. Transfer learning was employed using ImageNet (31) to overcome the limitations of small medical datasets, thereby enhancing generalization and accelerating model training. All architectures were implemented using the PyTorch 1.8.1 framework. Data augmentation was achieved through horizontal and vertical flipping and random cropping.

Thyroid ultrasound data were categorized into single-frame and five-frame multi-channel inputs for constructing the 2D static image model (DL_image) and the CEUS dynamic video model (DL_CEUSvideo), respectively. The single-frame input was based on the slice with the largest diameter of the thyroid nodule, whereas the five-frame input included this slice and two additional slices above and below at equal time intervals. The best-performing models from both the 2D static image and CEUS dynamic video models were selected for deep learning feature extraction, which was then combined to construct the DL_combined model (See **Supplementary Material** for model training details).

### 2.5 Interpretability of deep learning model results

To enhance the interpretability of the deep learning model’s predictions, Gradient-weighted Class Activation Mapping (Grad-CAM) was employed to generate heat maps that display the areas most indicative of OLNM in the images. This technique involves applying global average pooling to the model’s final feature map, calculating the gradient of the top-class output with respect to the final feature map, and subsequently visualizing this gradient on the original image (32).

### 2.6 Statistical analysis

The area under the receiver operating characteristic curve (AUC) was used to assess the efficacy of the deep learning models in predicting OLNM in PTC patients. The DeLong test was employed for model comparisons. Additionally, sensitivity, specificity, positive predictive value, negative predictive value, and accuracy were calculated to evaluate and compare the diagnostic performance of the models. A P-value of less than 0.05 was considered statistically significant. All statistical analyses were conducted using Python software (version 3.7.12) and the Statsmodels package (version 0.13.2).

## 3 Results

### 3.1 Clinical ultrasound characteristics

In the training set, ultrasound images from 280 PTC cases were analyzed, with an average age of 42.31 ± 10.97 years, comprising 70 males and 210 females. The average tumor diameter was 11.74 ± 7.06 mm. Moreover, 55 cases had OLNM, whereas 225 did not. The test set included 116 patients, with an average age of 40.70 ± 10.58 years, including 33 males and 83 females. Similarly, the average tumor diameter was 11.43 ± 7.02 mm. A total of 27 cases developed OLNM, whereas 89 did not. The analysis revealed significant differences in the prediction of PTC occult lymph node metastasis based on nodule maximum diameter, nodule margins, extracapsular extension, and CEUS peak intensity (P<0.05) (**Table 1**). There were no statistical differences in baseline characteristics between the training and test sets (**Supplementary Table S1**).

**Table 1:** Clinical patient characteristics and US features in the training set and test set.

Characteristics And US features	Training set (n=280)		P value	Test set (n=116)		P value
	OLNM- (n=225)	OLNM+ (n=55)		OLNM- (n=89)	OLNM+ (n=27)	
Age	42.84 ± 10.97	40.13 ± 10.81	0.107	41.72 ± 10.30	37.33 ± 11.01	0.059
Size	10.77 ± 6.30	15.69 ± 8.55	**<0.001**	10.45 ± 6.77	14.67 ± 6.98	**0.001**
Gender			0.019			0.17
Male	49 (21.78)	21 (38.18)		22 (24.72)	11 (40.74)	
Female	176 (78.22)	34 (61.82)		67 (75.28)	16 (59.26)	
Location			0.449			0.448
Upper	55 (24.44)	9 (16.36)		26 (29.21)	4 (14.81)	
Mid	105 (46.67)	29 (52.73)		41 (46.07)	14 (51.85)	
Lower	56 (24.89)	13 (23.64)		18 (20.22)	8 (29.63)	
sthmus	9 (4.00)	4 (7.27)		4 (4.49)	1 (3.70)	
Composition			>0.99			0.493
Solid	215 (95.56)	53 (96.36)		87 (97.75)	25 (92.59)	
Predominately solid	10 (4.44)	2 (3.64)		2 (2.25)	2 (7.41)	
Echogenicity			0.399			>0.99
Hypoechoic	217 (96.44)	54 (98.18)		88 (98.88)	27 (100.00)	
Isoechoic	6 (2.67)	0		1 (1.12)	0	
Hyperechoic	2 (0.89)	1 (1.82)		0	0	
Echotexture			>0.99			>0.99
Heterogeneous	225 (100.00)	55 (100.00)		89 (100.00)	27 (100.00)	
Orientation			0.151			>0.99
Horizontal	125 (55.56)	24 (43.64)		46 (51.69)	14 (51.85)	
Vertical	100 (44.44)	31 (56.36)		43 (48.31)	13 (48.15)	
Echogenic_foci			0.054			0.131
No	73 (32.44)	11 (20.00)		34 (38.20)	5 (18.52)	
Microcalcifications	138 (61.33)	43 (78.18)		54 (60.67)	22 (81.48)	
Macrocalcifications	14 (6.22)	1 (1.82)		1 (1.12)	0	
Margin			**<0.001**			**<0.001**
Ill-defined	80 (35.56)	51 (92.73)		25 (28.09)	27 (100.00)	
Irregular margin	145 (64.44)	4 (7.27)		64 (71.91)	0	
ETE			**<0.001**			**<0.001**
No	225 (100.00)	22 (40.00)		88 (98.88)	12 (44.44)	
Yes	0	33 (60.00)		1 (1.12)	15 (55.56)	
Halo			0.399			0.267
Present halo	7 (3.11)	0		1 (1.12)	2 (7.41)	
Absent halo	218 (96.89)	55 (100.00)		88 (98.88)	25 (92.59)	
CDFI			0.143			0.703
Avascularity	12 (5.33)	0		5 (5.62)	3 (11.11)	
Mainly peripheral vascularity	187 (83.11)	44 (80.00)		78 (87.64)	22 (81.48)	
Mainly central vascularity	4 (1.78)	2 (3.64)		1 (1.12)	0	
Mixed vascularity	22 (9.78)	9 (16.36)		5 (5.62)	2 (7.41)	
Enhancement_pattern			0.717			>0.99
Homogeneous	4 (1.78)	0		1 (1.12)	0	
Heterogeneous	221 (98.22)	55 (100.00)		88 (98.88)	27 (100.00)	
Peak_intensity			**0.019**			**0.018**
Hypoenhancement	157 (69.78)	48 (87.27)		63 (70.79)	26 (96.30)	
Isoenhancement	53 (23.56)	7 (12.73)		19 (21.35)	0	
Hyperenhancement	15 (6.67)	0		7 (7.87)	1 (3.70)	
Ring_enhancement			0.738			>0.99
Absent	221 (98.22)	53 (96.36)		88 (98.88)	27 (100.00)	
Present	4 (1.78)	2 (3.64)		1 (1.12)	0	
Nodule_composition_ at_CEUS			0.874			0.883
Solid	209 (92.89)	51 (92.73)		83 (93.26)	26 (96.30)	
Predominately solid	15 (6.67)	4 (7.27)		4 (4.49)	1 (3.70)	
Predominately cystic	1 (0.44)	0		1 (1.12)	0	
Cystic	0	0		1 (1.12)	0	

ETE, Extrathyroidal extension; CDFI, Color Doppler flow imaging; CEUS, Contrast-enhanced ultrasound; US, Ultrasound; OLNM, Occult lymph node metastasis; Mid, Middle.

The bolded values in Table 1 indicate p-values < 0.05 for easier identification.

### 3.2 Construction and selection of the optimal models for DL_image, DL_CEUSvideo, and DL_combined, respectively

**Figure 3:**
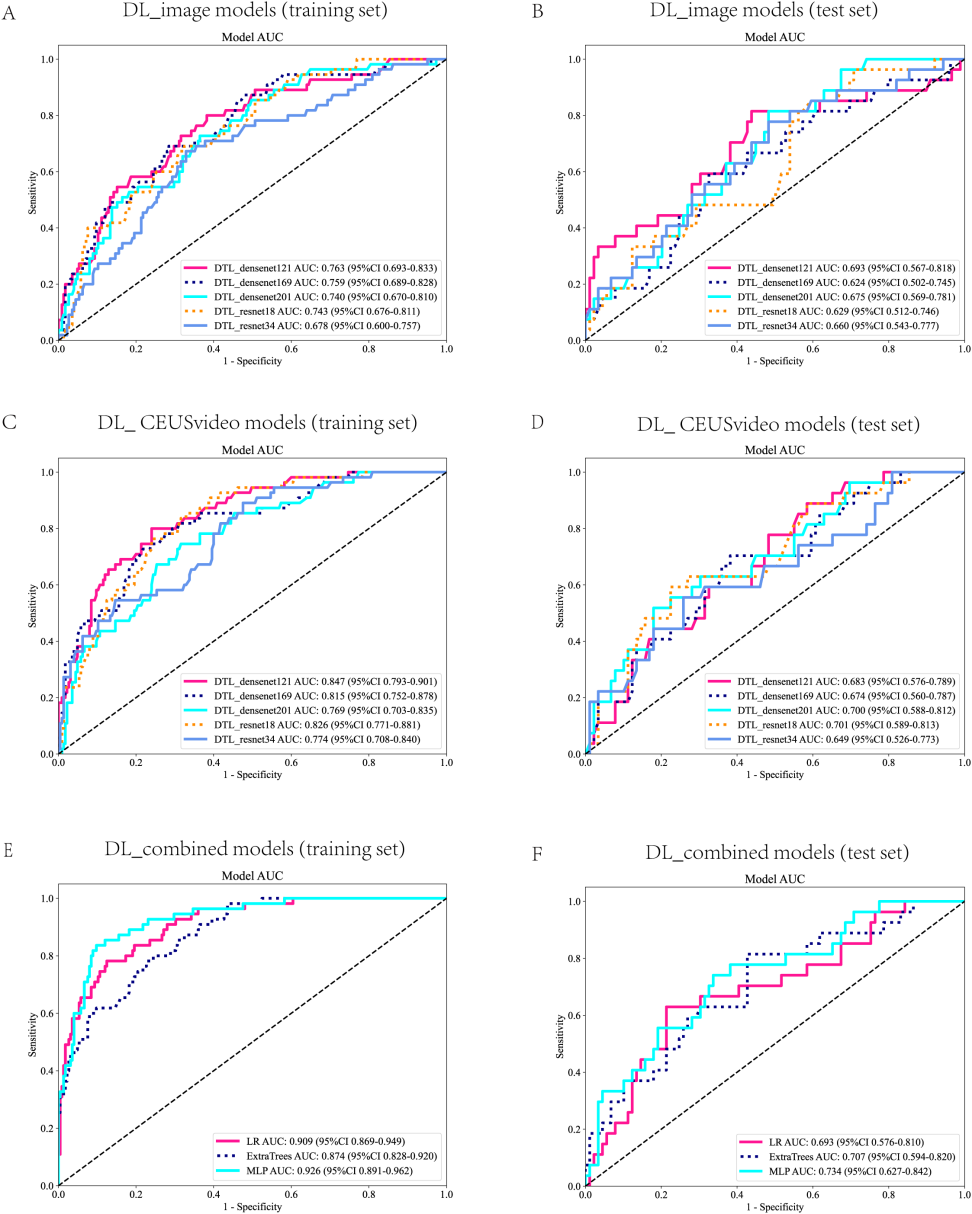
Receiver Operating Characteristic (ROC) Curves for the DL Models. ROC curves for the DL_image model in the training set **(A)** and test set **(B)**. ROC curves for the DL_CEUSVideo model in the training set **(C)** and test set **(D)**. ROC curves of the DL_Combined model in the training set **(E)** and test set **(F)**. ROC, Receiver Operating Characteristic; DL, Deep learning; CEUS, Contrast-enhanced ultrasound.

In this study, the DL_image and DL_CEUSvideo models were successfully constructed. Regarding the DL_image model, the DenseNet169 architecture demonstrated superior predictive performance in the test set (AUC = 0.624, 95% CI: 0.502–0.745) (**Figures 3A, B**). In contrast, among the DL_CEUSvideo model, the ResNet18 architecture showed superior predictive performance (AUC = 0.701, 95% CI: 0.589–0.813) (**Figures 3C, D**). Subsequently, the best-performing models, DenseNet169 (from DL_image) and ResNet18 (from DL_CEUSvideo), were selected to extract DL features for fusion and construction of the DL_combined model. In the DL_combined model, the MLP architecture achieved the highest predictive performance in the test set (AUC = 0.734, 95% CI: 0.627–0.842) (**Figures 3E, F**). Overall, the optimal models for predicting OLNM in PTC across the three approaches were DenseNet169, ResNet18, and MLP, respectively.

### 3.3 Comparison of predictive performance among DL_CEUSvideo, DL_image, and DL_combined models

**Figure 4:**
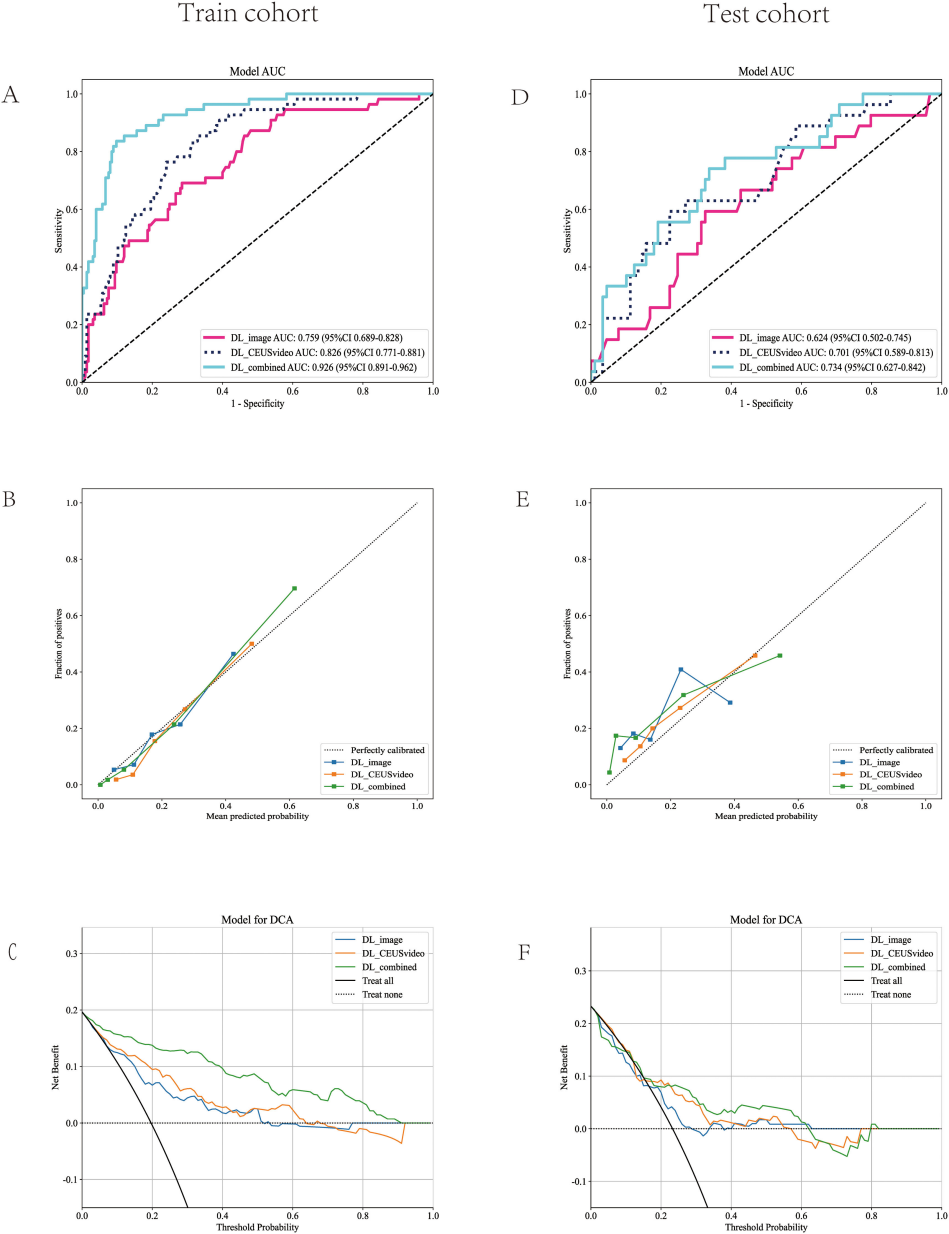
Model performance evaluation in the training **(A-C)** and test **(D-F)** cohorts. **(A, D)** ROC curves of five predictive models. **(B, E)** Calibration curves comparing predicted vs. actual probability across all models. **(C, F)** DCA demonstrates the clinical utility of each model. The ROC curve demonstrated that the DL_combined model achieved the highest AUC value. The calibration curve indicated that the predicted probabilities of the DL_combined model were in closer agreement with the actual probabilities. Decision curve analysis revealed that the DL_combined model provided higher clinical net benefit. ROC, Receiver Operating Characteristic; AUC, Area under the curve; DCA, Decision curve analysis; DL, Deep learning.

Furthermore, this study compared the performance of the best-performing models constructed using the CEUS video DL model, 2D static image DL model, and combined models, termed DL_CEUSvideo, DL_image, and DL_combined, respectively, for predicting OLNM in PTC patients. In the training set, the AUC values for the DL_CEUSvideo, DL_image, and DL_combined models were 0.826 (95% CI: 0.771-0.881), 0.759 (95% CI: 0.690-0.828), and 0.926 (95% CI: 0.891-0.962), respectively (**Figure 4A**). In the test set, the corresponding AUC values were 0.701 (95% CI: 0.589-0.813), 0.624 (95% CI: 0.502-0.745), and 0.734 (95% CI: 0.627-0.842), respectively (**Figure 4D**).

In the training set, the sensitivity, specificity, and accuracy of the DL_CEUSvideo, DL_image, and DL_combined models were 0.836, 0.671, and 0.704; 0.673, 0.716, and 0.707; and 0.818, 0.902, and 0.886, respectively. In the test set, these metrics were 0.556, 0.775, and 0.724; 0.556, 0.674, and 0.647; and 0.704, 0.663, and 0.672, respectively (**Table 2**).

**Table 2:** Comparison of the performance of the DL_CEUSvideo, DL_image, and DL_combined models in predicting OLNM in patients with PTC.

Model	AUC (95% CI)	Sensitivity	Specificity	PPV	NPV	Accuracy	Cohort
DL_image	0.759 (0.690 - 0.828)	0.673	0.716	0.366	0.899	0.707	Train
DL_CEUSvideo	0.826 (0.771 - 0.881)	0.836	0.671	0.383	0.944	0.704	Train
DL_combined	0.926 (0.891 - 0.962)	0.818	0.902	0.672	0.953	0.886	Train
DL_image	0.624 (0.502- 0.745)	0.556	0.674	0.341	0.833	0.647	Test
DL_CEUSvideo	0.701 (0.589 - 0.813)	0.556	0.775	0.429	0.852	0.724	Test
DL_combined	0.734 (0.627 - 0.842)	0.704	0.663	0.388	0.881	0.672	Test

DL, Deep learning; AUC, Area under the curve; CI, Confidence interval; PPV, Positive predictive value; NPV, Negative predictive value; CEUS, Contrast-enhanced ultrasound; OLNM, Occult lymph node metastasis; PTC, Papillary thyroid carcinoma.

Furthermore, model performance was assessed using calibration curves, which delineated that the DL_CEUSvideo, DL_image, and DL_combined models displayed satisfactory calibration in both training and test sets, as depicted in **Figures 4B, E**. Additionally, decision curve analysis (DCA) indicated that the DL_CEUSvideo and DL_combined models offered greater clinical benefit for the preoperative prediction of OLNM in PTC patients. The DCA for the DL_CEUSvideo, DL_image, and DL_combined models is delineated in **Figures 4C, F**.

### 3.4 Grad-CAM heatmap visualization

**Figure 5:**
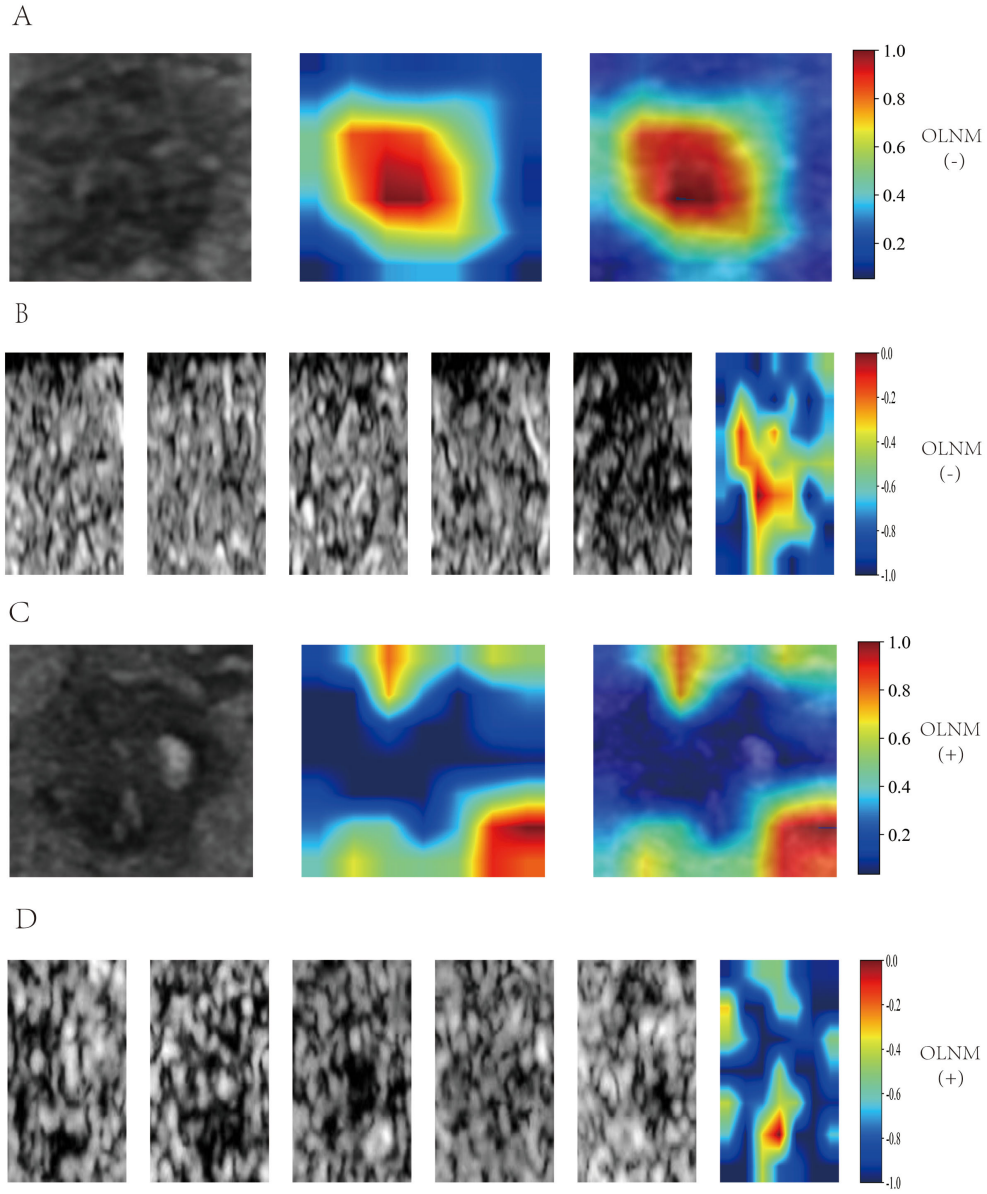
Grad-CAM Visualization of DL_image and DL_CEUSvideo Models. Single-frame heatmap visualization is labeled from 0.2 to 1.0, multi-frame heatmap visualization is labeled from -1.0 to 0, with positive values indicating OLNM positivity and negative values indicating OLNM negativity. In OLNM-positive ultrasound images, the response area is usually located at the edge of the tumor(C, D). In contrast, in OLNM-negative images, the response area is generally evenly distributed within the tumor(A, B). Grad-CAM, Gradient­weighted Class Activation Mapping; DL, Deep learning; CEUS, Contrast-enhanced ultrasound; OLNM, Occult lymph node metastasis.

Grad-CAM was utilized to generate heatmaps to visualize the recognition patterns of the deep transfer learning models. Heatmap visualizations for both the five-frame CEUS dynamic Videos and single-frame static images were created for OLNM-negative (**Figures 5A, B**) and OLNM-positive patients (**Figures 5C, D**). As anticipated, in most OLNM-positive ultrasound images, the response regions were typically located at the tumor margins. On the other hand, in OLNM-negative images, the response regions were generally distributed evenly within the tumors.

## 4 Discussion

In recent years, significant advances in deep learning have enabled machines to learn and process multi-scale, multi-level abstract data (33, 34) and automatically analyze and interpret complex datasets. Herein, the DL_CEUSvideo, DL_image, and DL_combined models were successfully developed to predict OLNM in PTC patients. The DL_CEUSvideo model demonstrated superior performance in predicting OLNM in PTC patients compared to the DL_image model, which was based on single-frame typical ultrasound images, achieving favorable results in the test set. These findings collectively validate the feasibility of using multimodal ultrasound dynamic video deep learning models to predict OLNM in PTC patients.

Of note, ultrasound is the preferred method for preoperative evaluation of cervical lymph node status. However, due to the unique anatomy of cervical lymph nodes, particularly the complex structure of central lymph nodes obscured by the esophagus, trachea, and mediastinal regions, ultrasound is less effective in detecting central lymph nodes compared to lateral ones (9, 14). Thus, timely and accurate preoperative prediction of central lymph node status is crucial. Earlier studies have identified tumor location, size (>5mm), microcalcifications, and extrathyroidal extension as independent predictors of OLNM (2, 16, 35). A recent study (36) using CEUS for lymph node evaluation described its accuracy in assessing LNM in PTC patients as unsatisfactory. Although these studies evaluated the utility of conventional ultrasound features for predicting OLNM in PTC patients, they relied on subjective visual assessments based on personal expertise and experience, inevitably leading to the loss of key information and reduced predictive performance. With advancements in artificial intelligence, deep learning surpasses traditional two-dimensional ultrasound by identifying subtle textures and details overlooked by radiologists, thus demonstrating superior diagnostic performance (20, 37).

Herein, the DL_image model achieved an AUC of 0.759 (95% CI: 0.690-0.828) in the training set and 0.624 (95% CI: 0.502-0.745) in the test set, consistent with the findings of previous studies (26, 38, 39). While it aids in improving the detection rate of OLNM in preoperative PTC patients, the results remain suboptimal. This may be ascribed to single-frame static images, merely representing a fraction of the tumor and the high heterogeneity of PTC tumors. Indeed, a single image cannot fully capture tumor heterogeneity and microenvironment changes. Extracting multiple keyframes from the CEUS dynamic video of the primary PTC lesion and using multi-channel inputs in the deep learning model effectively addressed the loss of key information from single static images, thereby achieving superior predictive performance. The DL_CEUSvideo model achieved an AUC of 0.826 (95% CI: 0.771-0.881) in the training set and 0.701 (95% CI: 0.589-0.813) in the test set, demonstrating outstanding predictive performance and improving OLNM detection rates in PTC patients preoperatively. Notably, the AUC was in line with that of previous studies (40, 41) on 2D image dynamic videos, despite being marginally lower than Zhao HN’s study (18), which focused on CEUS video based lymph node lesions. This discrepancy may be attributed to studies based on lymph node lesions being more direct, thereby providing more valuable key information. However, this study focused on predicting OLNM, and the lymph nodes of PTC patients typically do not manifest abnormalities during the preoperative period, leading to prediction models based on features derived from the primary lesion. Nevertheless, satisfactory predictive performance was achieved.

Interestingly, the DL_CEUSvideo and DL_image models exhibited low sensitivity, at 0.556, for predicting OLNM in PTC patients in the test set. This may be due to imprecise labeling of training data or a lack of data augmentation during training. To address this shortcoming, feature fusion was employed by integrating DL features extracted from both models to construct the DL_combined model, which achieved a sensitivity and specificity of 0.818 and 0.902, respectively, in the training set, and 0.704 and 0.663, respectively, in the test set. Compared to either individual models, sensitivity was significantly higher in the DL_combined model (0.704 vs. 0.556) without compromising specificity. Importantly, this finding indicates that the DL_combined model, through deep learning feature fusion, can overcome the limitations of individual models and enhance predictive performance. Thus, developing deep learning models from dynamic video can effectively optimize OLNM prediction in PTC patients, offering a novel, non-invasive, and accurate preoperative predictive method that can assist in personalized treatment decision-making and benefit patients.

To overcome the “black box” nature of deep learning models (42), Grad-CAM was utilized to develop heatmaps, allowing better interpretation of the decision-making process. Through the observation of Grad-CAM visualization results, it was found that when analyzing the aggressiveness of thyroid nodules, the deep learning model autonomously assigned greater weight to the solid components of the nodules. This finding is highly consistent with clinicians’ prioritization of solid components in clinical practice. The echogenicity, margin characteristics, morphological features, and calcification status of the solid components constitute critical radiological characteristics for diagnosing thyroid nodule aggressiveness (43). This phenomenon indicates that the optimized deep learning video model can, to some extent, effectively simulate clinicians’ diagnostic reasoning patterns. Interestingly, it was also observed that for thyroid nodules with calcifications, the model intelligently assigned higher weight to calcified regions, which aligns with the findings reported by Zhang C et al. (44). Previous studies have confirmed that different types of echogenic foci observed in ultrasound imaging, including microcalcifications, coarse calcifications, and peripheral calcifications, correlate well with the probability of thyroid nodule malignancy (43, 44). Herein, the response areas were typically located at the tumor margins in the ultrasound images of most OLNM-positive patients. In contrast, in OLNM-negative images, the response areas were generally evenly distributed within the tumor. This signals that the DL model primarily focused on areas of interest similar to those evaluated by clinicians to predict OLNM, thereby enhancing the interpretability of the deep learning model, reinforcing the credibility of the model training process, and increasing clinicians’ confidence in adopting these models for decision-making.

Currently, the management of cervical lymph node metastasis in preoperative lymph node-negative PTC remains controversial, especially concerning the need for intraoperative prophylactic central neck lymph node dissection (CLND). The clinical challenge is to limit the risk of surgical complications from CLND while minimizing recurrence and poor prognosis associated with central neck lymph node metastasis in PTC patients. Mounting evidence suggests that surgery is no longer the sole treatment option for malignant nodules, with local ablation therapy being an alternative modality for appropriately selected patients (45, 46). However, failure to accurately identify OLNM preoperatively may lead to recurrence and unfavorable prognosis (47). The developed DL_CEUSvideo and DL_combined models can predict OLNM in PTC patients preoperatively through non-invasive examination, potentially identifying a larger number of OLNM cases. This further highlights the clinical value of the DL video models for predicting OLNM in PTC patients.

Nevertheless, this study has some limitations that merit acknowledgment. Firstly, as a retrospective study, the results relied on limited retrospectively collected data, which may introduce inherent bias. Future studies should include more modalities and adopt prospective designs to enhance the diagnostic performance of the model. Secondly, although all ultrasound examinations were performed by experienced physicians, intra-operator variability in image acquisition might have compromised image consistency. Lastly, this was a two-center study that lacked sufficient external validation. Future studies are warranted to collect multi-center data for external validation to enhance the generalizability and robustness of the model.

In summary, this study demonstrated that the multimodal deep-learning video model can accurately predict OLNM status in PTC patients. The DL_CEUSvideo model outperformed the DL_image model, while the DL_combined model addressed the limitations of single models, further improving predictive performance and concomitantly increasing OLNM detection rates in PTC patients. This novel approach has the potential to serve as an effective alternative for preoperative OLNM screening in clinically lymph node-negative PTC patients and aid in clinical decision-making.

## Data Availability

The original contributions presented in the study are included in the article/**Supplementary Material**. Further inquiries can be directed to the corresponding authors.
