# Supplementary material for: A novel deep learning model based on multimodal contrast-enhanced ultrasound dynamic video for predicting occult lymph node metastasis in papillary thyroid carcinoma
Source: Front Endocrinol (Lausanne). 2025 Jul 24;16:1634875. doi: 10.3389/fendo.2025.1634875 (PMC12329689; doi:10.3389/fendo.2025.1634875)
Supplement: Supplementary file 1 [file DataSheet1.pdf]

Supplementary Material

1 Supplementary Data

1.1 Detailed description of the training process in this study

1.1.1 Data preprocessing

Following routine two-dimensional ultrasound and CEUS examinations, the highest-quality video recording and single-frame images with minimal artifacts were selected for analysis. All thyroid ultrasound dynamic videos extracted from the system were converted to and stored in Audio Video Interleave (AVI) format. Upon obtaining the raw thyroid CEUS dynamic video data, the nodule region was extracted. Next, a physician with over five years of experience in thyroid CEUS reviewed the video to determine nodule size and boundaries, ensuring image quality. Using FFmpeg software (version 4.0.2), the original AVI files were processed, and thyroid CEUS videos (lasting approximately 10-120 seconds) were downsampled at a rate of one frame per two seconds to extract keyframes. For each video, 1-15 frames with clear nodule features were selected, and the final five keyframes were selected at equal time intervals. This method reduced the need for ultrasound specialists to delineate all nodule regions in the video, as minimal variation exists between adjacent frames within a two-second interval, allowing efficient capture of key nodule features.

Thereafter, the extracted video data images were preprocessed and normalized. Steps included: (1) Retaining only the central lesion area by cropping and excluding irrelevant edge information; (2). Grayscale conversion was performed (as ultrasound images are inherently monochromatic). (3). Annotations (including measurement scales and calipers) were removed to prevent potential model bias ("answer peeking"). (4). Resizing ultrasound images to 256×256 pixels and applying data augmentation methods such as random flipping and cropping to enhance data diversity, model generalization, and robustness, thereby mitigating the risk of overfitting; (5). Scaling ultrasound images to 224×224 pixels and normalizing pixel values to a range of 0 to 1.

Data Augmentation Strategies (applying minor perturbations to images, with variations possible for each input based on practical conditions): (1) Random rotation: ±25°; (2). Shear: ±10°; (3). Translation: Up to 5% of the image dimensions; (4). Brightness adjustment: ±20%; (5). Contrast adjustment: ±20%; (f). Random horizontal flipping (probability = 0.5).

1.1.2 Development of 2D static images, CEUS dynamic video DL models, and DL_combined model

In this study, five representative deep learning architectures were employed for pretraining, namely DenseNet121, DenseNet169, DenseNet201, ResNet18, and ResNet34. To address the limitation of small-scale medical imaging datasets, a transfer learning strategy based on ImageNet was adopted to enhance model generalization capability and accelerate the training process. All architectures were implemented using the PyTorch 1.8.1 framework. The pretrained weights from ImageNet were loaded for each network architecture, with an initial learning rate of 0.01. The cross-entropy loss function was utilized, and the models were trained for 50 epochs with a dropout rate of 0.2. The model parameters achieving the highest accuracy on the test set were retained. Stochastic gradient descent (SGD) was selected as the optimizer.

1.1.2.1 2D static images DL Signature

1.1.2.1.1 The largest ROI section in the imaging data was cropped to form roi_images. Based on transfer learning, a model was trained, and features were extracted to obtain deep learning features.

1.1.2.1.2 Ultrasound images in the training and testing sets were cropped according to the defined ROI, with a unified size adjustment to 224 × 224. The cropped ROI images were then randomly divided into a training set (70%) and a testing set (30%).

1.1.2.1.3 Features were extracted from ROI images using the second-to-last layer of the model, namely the avgpool layer.

1.1.2.1.4 The avgpool layer contained 2048 features, which were reduced to 100 dimensions using principal components analysis (PCA).

1.1.2.1.5 The features were normalized (Z-score) to transform the data to follow a normal distribution N~(0, 1).

1.1.2.1.6 Various machine learning algorithms, including random forest (RF), k-nearest neighbors (KNN), logistic regression (LR), extreme gradient boosting (XGBoost), multilayer perceptron （MLP）, and Extra Trees, were used to train the data on the development set.

1.1.2.2. CEUS dynamic video DL Signature

1.1.2.2.1 Multi-scale-based transfer learning algorithm for slice fusion.

1.1.2.2.2 A transfer learning algorithm was employed to train the deep learning model. A fine-tuned model was obtained and subsequently applied to predict the probabilities for all cropped slices.

1.1.2.2.3 The PAtterns of LIkeness (PALI) method is applied for histogram statistics of all predicted probabilities, while the Bag of Words (BoW) algorithm is used for Term Frequency-Inverse

Document Frequency (TF-IDF) feature extraction, yielding corresponding features from both approaches.

1.1.2.2.4 Machine learning algorithms, including random forest (RF), k-nearest neighbors (KNN), logistic regression (LR), extreme gradient boosting (XGBoost), multilayer perceptron（MLP）, and Extra Trees, were separately applied to model the features obtained from the two pathways.

1.1.2.2.5 The final prediction results were derived by fusing (averaging) the outputs from both pathways.

1.1.2.3 DL_combined model construction

The DenseNet169 and ResNet18 models, which demonstrated optimal performance in the 2D static image model (DL_image) and the CEUS dynamic video model (DL_CEUSvideo), were selected for feature extraction. The extracted DL features were subsequently integrated through deep learning-based feature fusion to construct a comprehensive model. Among the evaluated integrated models, multilayer perceptron (MLP), logistic regression (LR), and ExtraTrees exhibited superior performance. By combining the deep transfer learning features extracted from both the DL_image and DL_CEUSvideo models, a stacking ensemble method is employed to integrate the model metrics, ultimately constructing a comprehensive prediction model.

1.2 The calculation formula of the performance metrics

1.2.1 Area Under the Curve (AUC): AUC represents the area beneath the receiver operating characteristic curve (ROC), which reflects the classification ability of a model across various thresholds. The AUC value ranges from 0 to 1, with higher values indicating better classification performance of the model. AUC comprehensively considers both the true positive rate (sensitivity) and false positive rate (1-specificity) of the model, providing an overall performance evaluation that is suitable for situations involving unbalanced datasets.

1.2.2 Accuracy: Accuracy measures the proportion of all correctly identified instances over the total instances. It is calculated as:

$$Accuracy = \frac{\mathrm{TP} + \mathrm{TN}}{\mathrm{TP} + \mathrm{TN} + \mathrm{FP} + \mathrm{FN}}$$

Where *TP* is the true positive instances, *TN* is the true negative instances, *FP* is the false positive instances, and *FN* is the false negative instances.

Accuracy serves as the most intuitive evaluation metric, suitable for scenarios where the distribution of sample categories is relatively balanced. However, in cases of unbalanced datasets, accuracy can be misleading, thus it should be used in conjunction with other metrics.

1.2.3 Sensitivity/Recall: Sensitivity, also known as recall, indicates the model's ability to correctly identify positive samples. Sensitivity measures the proportion of actual positives that are correctly identified by the model. It is calculated as:

$$Sensitivity = \frac{\mathrm{TP}}{TP + FN}$$

In medical diagnosis, missed detections (false negatives) can lead to severe consequences, making sensitivity a crucial metric, especially in situations where minimizing missed detections is essential, such as tumor screening.

1.2.4 Specificity: Specificity measures the proportion of actual negatives that are correctly identified by the model. It is calculated as:

$$Specificity = \frac{TN}{TN + FP}$$

It measures the false positive rate, providing insights into the model's performance in recognizing negative samples. In certain clinical contexts, false positives can result in unnecessary treatments or anxiety, highlighting the importance of specificity.

1.2.5 Positive Predictive Value (PPV): The PPV represents the proportion of samples predicted as positive that are actually positive. It is calculated as:

$$\mathrm{PPV} = \frac{\mathrm{TP}}{\mathrm{TP} + \mathrm{FP}}$$

The PPV measures the accuracy of a model, particularly in clinical decision-making where doctors need to understand the reliability of positive results.

1.2.6 Negative Predictive Value (NPV): The NPV indicates the proportion of samples predicted as negative that are actually negative. It is calculated as:

$$NPV = \frac{\mathrm{TN}}{\mathrm{TN} + \mathrm{FN}}$$

The NPV is equally important, providing critical information when evaluating model performance, especially when ensuring the reliability of negative results.

1.2.7 F1-score: The F1-score is the harmonic mean of precision and recall, It is calculated as:

$$F1 - score = \frac{2 * (\mathrm{accuracy} \ * \ \mathrm{recall})}{(\mathrm{accuracy} \ + \ \mathrm{recall})}$$

The F1 score is particularly useful when dealing with unbalanced datasets, allowing a balance between precision and sensitivity. Through the F1-score, researchers can evaluate the overall performance of the model in classifying both positive and negative samples.

1.3 Detailed description of CEUS parameters

In this study, CEUS was performed using L2-9 (frequency range: 2-9 MHz) and ML6-15 (frequency range: 6-15 MHz) linear array transducers with a mechanical index of 0.12. The scanning frame rate was set at 15 frames per second to optimize image quality. The contrast agent was administered via intravenous bolus injection 1-2 seconds after initiating ultrasound scanning, and continuous observation was maintained for at least 3-5 minutes post-injection to ensure optimal image acquisition during the dynamic phase of tumor blood perfusion. A 2.4 mL dose of SonoVue contrast agent was injected through the antecubital vein, followed by a 5 mL saline flush, with simultaneous video recording initiation. The CEUS dynamic video storage protocol included: (1) capturing the complete scanning sequence from contrast agent appearance to washout; (2) ensuring comprehensive visualization of nodule morphology and surrounding tissue architecture during acquisition; and (3) maintaining a minimum recorded video duration of 10 seconds.

2. Supplementary Table

Table S1 Comparison of clinical characteristics and US features between training set and test set

Characteristics And US features	Total (n=396)	Test set (n=116)	Training set (n=280)	P value
**Age**	41.84±10.87	40.70±10.58	42.31±10.97	0.213
**Size(mm)**	11.65±7.04	11.43±7.02	11.74±7.06	0.625
**Gender**				0.558
Male	103(26.01)	33(28.45)	70(25.00)	
Female	293(73.99)	83(71.55)	210(75.00)	
**location**				0.918
Upper	94(23.74)	30(25.86)	64(22.86)	
Mid	189(47.73)	55(47.41)	134(47.86)	
Lower	95(23.99)	26(22.41)	69(24.64)	
Sthmus	18(4.55)	5(4.31)	13(4.64)	
**Composition**				0.917
Solid	380(95.96)	112(96.55)	268(95.71)	
Predominately solid	16(4.04)	4(3.45)	12(4.29)	
**Echogenicity**				0.359
Hypoechoic	386(97.47)	115(99.14)	271(96.79)	
Isoechoic	7(1.77)	1(0.86)	6(2.14)	
Hyperechoic	3(0.76)	0	3(1.07)	
**Echotexture**				> 0.99
Heterogeneous	396(100.00)	116(100.00)	280(100.00)	
**Orientation**				0.873
Horizontal	209(52.78)	60(51.72)	149(53.21)	
Vertical	187(47.22)	56(48.28)	131(46.79)	
**Echogenic_foci**				0.108
No	123(31.06)	39(33.62)	84(30.00)	
Microcalcifications	257(64.90)	76(65.52)	181(64.64)	
Macrocalcifications	16(4.04)	1(0.86)	15(5.36)	
**Margin**				0.806
Ill-defined	183(46.21)	52(44.83)	131(46.79)	
Irregular margin	213(53.79)	64(55.17)	149(53.21)	
**ETE**				0.701
No	347(87.63)	100(86.21)	247(88.21)	
Yes	49(12.37)	16(13.79)	33(11.79)	
**Halo**				> 0.99
Present halo	10(2.53)	3(2.59)	7(2.50)	
Absent halo	386(97.47)	113(97.41)	273(97.50)	
**CDFI**				0.244
Avascularity	20(5.05)	8(6.90)	12(4.29)	
Mainly peripheral vascularity	331(83.59)	100(86.21)	231(82.50)	
Mainly central vascularity	7(1.77)	1(0.86)	6(2.14)	
Mixed vascularity	38(9.60)	7(6.03)	31(11.07)	
**Enhancement_pattern**				> 0.99

Table S1 (continued)

Characteristics And US features	Total (n=396)	Test set (n=116)	Training set (n=280)	P value
Homogeneous	5(1.26)	1(0.86)	4(1.43)	
Heterogeneous	391(98.74)	115(99.14)	276(98.57)	
**Peak_intensity**				0.468
Hypoenhancement	294(74.24)	89(76.72)	205(73.21)	
Isoenhancement	79(19.95)	19(16.38)	60(21.43)	
Hyperenhancement	23(5.81)	8(6.90)	15(5.36)	
**Ring_enhancement**				0.645
Absent	389(98.23)	115(99.14)	274(97.86)	
Present	7(1.77)	1(0.86)	6(2.14)	
**Nodule_composition_at_CEUS**				0.300
Cystic	1(0.25)	1(0.86)	0	
Solid	369(93.18)	109(93.97)	260(92.86)	
Predominately solid	24(6.06)	5(4.31)	19(6.79)	
Predominately cystic	2(0.51)	1(0.86)	1(0.36)	

ETE: Extrathyroidal extension; CDFI: Color Doppler flow imaging; CEUS: Contrast-enhanced ultrasound US: Ultrasound; Mid: Middle
